# Human Mitochondrial DNA-Protein Complexes Attach to a Cholesterol-Rich Membrane Structure

**DOI:** 10.1038/srep15292

**Published:** 2015-10-19

**Authors:** Joachim M. Gerhold, Şirin Cansiz-Arda, Madis Lõhmus, Oskar Engberg, Aurelio Reyes, Helga van Rennes, Alberto Sanz, Ian J. Holt, Helen M. Cooper, Johannes N. Spelbrink

**Affiliations:** 1 Nijmegen Centre for Mitochondrial Disorders, RadboudUMC, Nijmegen, The Netherlands; 2 Faculty of Science and Engineering, Biochemistry. Åbo Akademi, Turku, Finland; 3 Institute of Molecular and Cell Biology, University of Tartu, Estonia; 4 MRC Mitochondrial Biology Unit, Cambridge, UK; 5 Institute for Cell and Molecular Biosciences, Newcastle University Institute for Ageing, University of Newcastle, Newcastle upon Tyne, NE4 5PL, UK; 6 MRC National Institute for Medical Research, London, NW7 1AA, UK

## Abstract

The helicase Twinkle is indispensable for mtDNA replication in nucleoids. Previously, we showed that Twinkle is tightly membrane-associated even in the absence of mtDNA, which suggests that Twinkle is part of a membrane-attached replication platform. Here we show that this platform is a cholesterol-rich membrane structure. We fractionated mitochondrial membrane preparations on flotation gradients and show that membrane-associated nucleoids accumulate at the top of the gradient. This fraction was shown to be highly enriched in cholesterol, a lipid that is otherwise low abundant in mitochondria. In contrast, more common mitochondrial lipids, and abundant inner-membrane associated proteins concentrated in the bottom-half of these gradients. Gene silencing of ATAD3, a protein with proposed functions related to nucleoid and mitochondrial cholesterol homeostasis, modified the distribution of cholesterol and nucleoids in the gradient in an identical fashion. Both cholesterol and ATAD3 were previously shown to be enriched in ER-mitochondrial junctions, and we detect nucleoid components in biochemical isolates of these structures. Our data suggest an uncommon membrane composition that accommodates platforms for replicating mtDNA, and reconcile apparently disparate functions of ATAD3. We suggest that mtDNA replication platforms are organized in connection with ER-mitochondrial junctions, facilitated by a specialized membrane architecture involving mitochondrial cholesterol.

Mitochondrial DNA (mtDNA) is associated with proteins forming complexes called nucleoids (for a review, see e.g.^1^). Nucleoids include proteins involved in mtDNA replication, such as the mitochondrial DNA helicase Twinkle and the mitochondrial DNA polymerase polG. Since the discovery of mutations in mtDNA and in its maintenance proteins with an accompanying accumulation of secondary mtDNA mutations^2^,^3^,^4^, the organization and segregation of mtDNA and nucleoid proteins has become a topic of active investigation, and significant advances have been made in understanding the composition and dynamics of human mitochondrial nucleoids.

Since nucleoids involve protein-DNA and protein-RNA interactions to facilitate mtDNA maintenance and gene expression they likely are very dynamic^5^. According to this view nucleoids exist in several populations, which differ in their protein components depending on functional requirements such as replication, translation and repair. Previous work has identified transcriptionally active subsets of nucleoids^6^, while we recently identified a subset, which we proposed constitutes membrane-associated replication platforms, based both on *in situ* analyses of replication dynamics and biochemical fractionations^7^. These nucleoids contain the major replication proteins (Twinkle, mtSSB and polG as well as other proteins) and are distinguished by being more tightly bound to the membrane than other mtDNA nucleoids that are largely devoid of, in particular, Twinkle^7^.

ATAD3A and the less abundant ATAD3B are protein paralogs. They likely form heterohexamers or homohexamers of ATAD3A, which extend from the IM into the outer mitochondrial membrane (OM)^8^,^9^ and will be collectively termed ATAD3 from here on. In a previous study we found that ATAD3 co-purified with mtDNA maintenance proteins as well as mitochondrial ribosomes^9^. Moreover, the altered expression of ATAD3 perturbed mtDNA topology, as well as mitochondrial protein synthesis^10^,^11^. These observations prompted us to propose that ATAD3 supports nucleoids and mitochondrial ribosomes on the IM^11^. In addition, ATAD3 co-purified with proteins involved in lipid metabolism^11^ and has been detected in mitochondria associated ER membranes (MAM)^12^, biochemical isolates believed to represent ER-mitochondrial junctions. ATAD3 has been shown to regulate intestinal fat storage in nematodes^13^ and cholesterol channeling in a steroidogenic cell line^14^. Taken together, these observations suggest that ATAD3 is involved in the biogenesis or structural maintenance of mitochondrial membranes. The different roles of ATAD3 can be reconciled by suggesting a function for ATAD3 in maintaining mitochondrial membrane organization with direct bearing on the organization of mtDNA nucleoids. ATAD3 may thus play an important role also in nucleoid segregation^15^.

Although the composition of the mitochondrial membranes has been described^16^ little is known about the lateral organisation or dynamics of their lipids. The role of cholesterol has been puzzling: at 0.1 the ratio of cholesterol to phospholipids in mitochondria is very low^17^. As a result, mitochondrial cholesterol is considered unable to form archetypal cholesterol-rafts, where cholesterol interacts with saturated sphingolipids^18^,^19^. And indeed in one study, no such structures were detected in mitochondrial membrane preparations^20^. Notwithstanding this, based on studies on cholesterol behaviour in model systems, the curvature of the IM could restrict the distribution of cholesterol, thus causing the formation of locally concentrated domains in areas of low curvature^21^. Also, it has been proposed that proteins that exhibit scaffold–like properties, such as those belonging to the stomatin/prohibitin/flotilin/HflK/C (SPFH) family, could spatially organize the lipids in the mitochondrial membranes and thus define functional membrane domains^22^,^23^.

The contact sites between mitochondria and the ER are hubs for lipid translocation and Ca^2+^ traffic between the ER and mitochondria (reviewed by^24^). It has also been demonstrated that these junctions mark the sites of division in the course of mitochondrial network dynamics^25^. Moreover, ER-associated mitochondrial division has been spatially linked to nucleoids in yeast^26^, and also in mammalian cells nucleoids have been observed to often lie adjacent to dynamin related protein 1 (Drp1)^27^,^28^, which controls mitochondrial fission.

Here, we fractionated mitochondrial membrane preparations from human embryonic kidney (HEK) cells on gradients under conditions which preserve detergent resistant membrane structures. As we have shown previously^7^ the membrane-associated mtDNA and bound nucleoid proteins were concentrated in a distinct fraction at the top of the gradient. We analysed the lipid distribution in the gradients and discovered that the majority of the mitochondrial cholesterol co-fractionated with the nucleoid. Gene silencing of ATAD3 in HEK cells disrupted the cholesterol-containing domain and the nucleoids. We also detected the MAM marker protein, FACL4 (also known as ACSL4)^29^ in the cholesterol containing nucleoid fraction, while *vice-versa* ATAD3 and nucleoid associated replication factors were evident in purified MAM preparations. Our findings suggest a model, in which mammalian replicating mtDNA-protein complexes are directly or indirectly connected to the ER via a cholesterol-rich membrane platform, whose organisation relies on ATAD3.

## Results

### The majority of the mitochondrial cholesterol co-fractionates with the membrane-associated nucleoid

**Figure 1:**
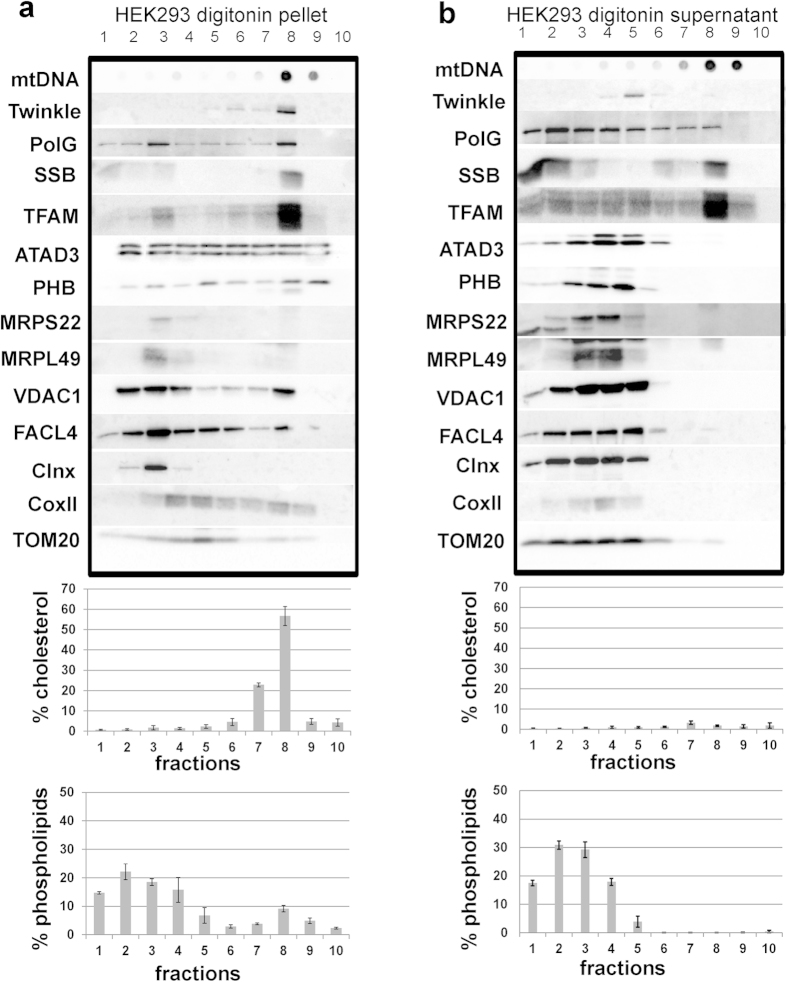
The majority of the cholesterol in the mitochondrial inner membrane co-fractionates with the membrane associated nucleoid. Pellet (**a**) and supernatant (**b**) preparations from digitonin treated purified mitochondria from HEK293 cells were separated on bottom-up flotation iodixanol gradients and their protein, DNA and lipid contents were analysed. The mtDNA and associated nucleoid proteins (Twinkle, polG, TFAM, SSB) as well as most of the cholesterol migrate in the same fraction in the pellet gradient. Some of the MAM marker protein FACL4 co-fractionates with the nucleoid in fraction 8. mtDNA, mitochondrial DNA; polG, polymerase Gamma; SSB, single-stranded binding protein; TFAM, mitochondrial transcription factor A; PHB, prohibitin, MRPS, mitochondrial ribosomal protein small subunit; MRPL, mitochondrial ribosomal protein large subunit; Clnx, calnexin; coxII, cytochrome oxidase subunit II; TOM20, translocase of outer membrane 20 kDa subunit; VDAC1, voltage-dependent anion channel 1. The lipid data is from the analyses of three replicate gradients (SEM), the protein and DNA profiles are from one representative gradient, while all three gradients were tested with at least the Twinkle, mtSSB, coxII, TFAM and ATAD3 antibodies by Western blot analysis and dot-blot analysis for mtDNA. The antibody probings were done on multiple blots (typically 2–3) with the same fractions to allow for the multiple detections with the many antibodies used.

We treated purified mitochondria from HEK293 cells with digitonin and centrifuged them to produce a mitochondrial membrane pellet fraction and a supernatant fraction containing soluble components. Both the pellet and the supernatant were next incubated with Triton X-100 on ice and fractionated on bottom-up Optiprep flotation gradients, where detergent resistant membrane structures and associated proteins, akin to lipid rafts, migrate close to the top of the gradient^30^. Previously, we showed that the pellet, obtained with the initial digitonin lysis of mitochondria, contained the majority of the helicase Twinkle and sub-sets of other replicative nucleoid proteins which, together with mtDNA, resolved in a discrete low density fraction in the gradient^7^. Here, we have repeated the flotation experiments, analyzed the lipid profile of the different fractions from such flotation gradients and show that the majority of the mitochondrial cholesterol is concentrated in the same fractions as the Twinkle-containing nucleoids (Fig. 1a and SFig. 1a). In the pellet these nucleoids and more than 50% of the cholesterol were concentrated in a single fraction. The membrane pellet as a whole contained the bulk of the total detected mitochondrial cholesterol (90%) and approximately 50% of the mitochondrial phospholipids (SFig. 1b,c). No major cholesterol peaks were seen in the supernatant gradient (Fig. 1b). The bulk of the common mitochondrial phospholipids, such as cardiolipin and phosphatidyl ethanolamine, was seen at the bottom of both gradients with a small peak associated with the cholesterol-containing fraction in the pellet gradient, mainly due to a moderate enrichment (compared to adjacent fractions) of phosphatidyl choline and an as yet unidentified other lipid species (SFig. 1a). In agreement with our previously published data^7^, in which we analyzed various proteins by Western blot in similar digitonin pellet and supernatant fractions, albeit without further gradient purifications, polG, mtSSB and TFAM are more or less equally present in both the pellet and supernatant flotation gradients, whereas Twinkle is abundant in particular in the pellet flotation gradient. Nonetheless, all these four nucleoid and replication proteins show a clear co-fractionation at low density with mtDNA and cholesterol, with Twinkle in particular migrating almost exclusively to the top of the gradient. ATAD3 shows a wide distribution over both gradients, but only in the digitonin pellet gradient does it also co-fractionate with the bulk of the mtDNA. CoxII, a mitochondrial inner membrane protein, is as expected mostly found in the pellet flotation, with the bulk migrating to a higher density together with the most abundant mitochondrial phospholipids (see also^7^), whereas mitoribosomal proteins are more soluble with most fractionating in the supernatant gradient at higher density than the bulk of the mtDNA. The outer membrane protein Tom20 is significantly enriched in the supernatant fraction, reflecting the higher sensitivity of the outer membrane to digitonin extraction and in agreement with our previous observations when testing various digitonin to protein ratios^7^. Our results thus reproduce and extend our previous demonstration that a Twinkle-containing nucleoid pool is membrane associated. We now further show the unusual co-fractionation of this membrane-associated nucleoid with most of the mitochondrial cholesterol in flotation gradients.

### Twinkle-cholesterol co-fractionation persists in the absence of mtDNA

**Figure 2:**
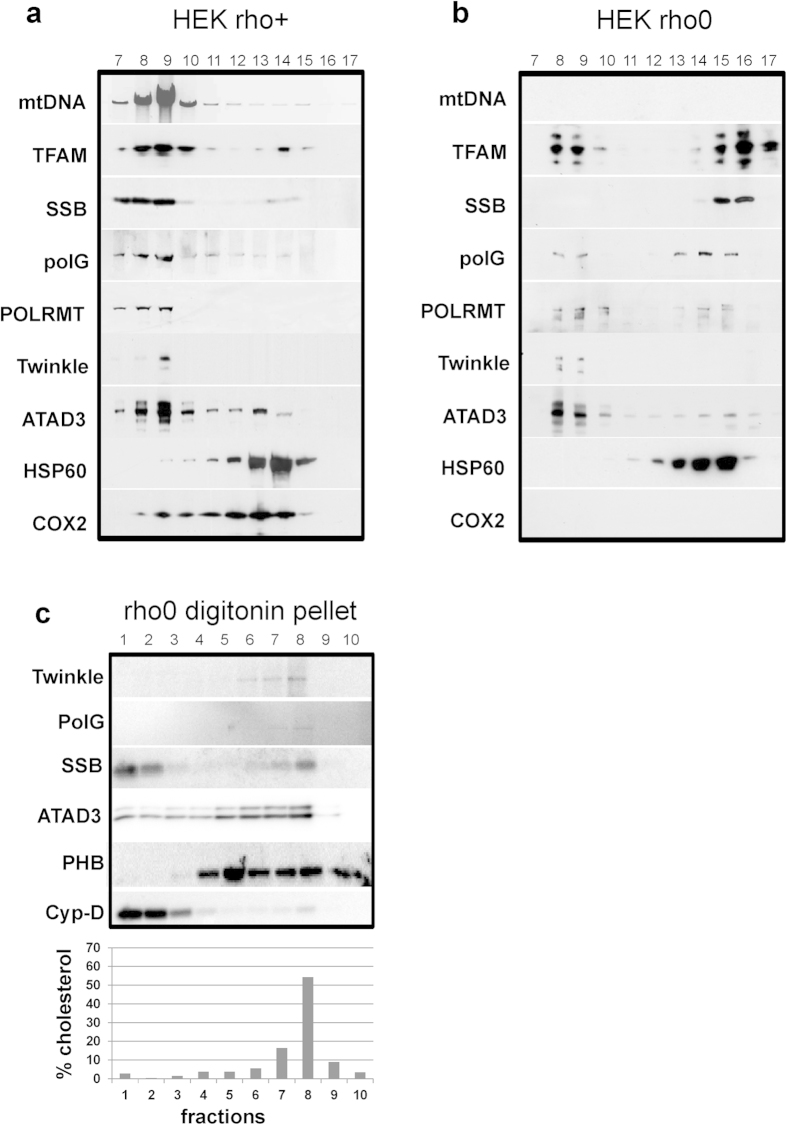
Nucleoid components and cholesterol co-sediment in the absence of mitochondrial DNA. Total mitochondrial lauryl-maltoside lysates from mtDNA containing control cells (**a**) and ρ° cells (**b**) were separated on top-down iodixanol gradients. Though some of the nucleoid component are lost or have moved to the top of the gradient in the absence of mtDNA, some still co-sediment at the bottom of the gradient. Pellet preparations from digitonin treated purified mitochondria from ρ° cells were separated on a bottom-up flotation iodixanol gradient (**c**). The nucleoid-protein and cholesterol fractions remain intact and co-fractionate also in the absence of mtDNA. mtDNA, mitochondrial DNA; TFAM, mitochondrial transcription factor A; SSB, single-stranded binding protein; polG, polymerase Gamma; POLRMT, mitochondrial RNA polymerase; HSP60, heat shock protein 60; coxII, cytochrome oxidase subunit II, PHB, prohibitin; Cyp-D, Cyclophilin D. Protein, DNA and lipid profiles of representative gradients.

Much can be learned about mtDNA metabolism by comparing cells that possess mtDNA with others that lack nucleic acids in their mitochondria, so-called ρ° cells. The strong association of Twinkle with a, perhaps specialized, membrane structure and the demonstration that also in ρ° cells Twinkle is found in distinct and discrete foci^3^,^7^,^27^ suggest that, similar to *S. cerevisiae* ρ° cells^31^, a remnant or minimal replication platform might exist in the absence of mtDNA in human cells. To test this hypothesis, we first prepared a lauryl-maltoside lysate from total mitochondria purified from mtDNA-containing HEK293 cells, and fractionated the lysate on a top-down iodixanol density gradient^11^. Established mitochondrial nucleoid and replication proteins were concentrated in the same fractions as the mtDNA (Fig. 2)^11^. Gradients using total mitochondrial lysates derived from ρ° cells (Fig. 2b) showed that DNA replication proteins become redundant and, as a result, in the ρ° gradients most of the remaining nucleoid proteins, but *not* Twinkle, relocated to the top of the iodixanol gradient (Fig. 2b, compare fractions 15,16,17 to Fig. 2a fractions 15,16,17 and 8,9,10). However, some of the remaining TFAM, polG and POLRMT still sedimented with Twinkle to the same fractions, in which the mtDNA resolved in samples from control cells (Fig. 2b fractions 8,9,10 and Fig. 2a). Moreover, also ATAD3 showed a clear co-fractionation with these remnant nucleoid proteins. This observation thus shows that several nucleoid proteins persist together in a relatively high-density iodixanol fraction and suggests, as in yeast, the presence of a minimal (membrane-associated) replisome in the absence of mtDNA. To further investigate the nature of the Twinkle-membrane association in ρ° cells, we next analysed digitonin-derived mitochondrial membranes from ρ° cells on bottom-up flotation Optiprep gradients. The lipid profile of the ρ° pellet digitonin fraction was found to be identical to that of its mtDNA-containing counterpart, including the abundance and buoyant density of the mitochondrial cholesterol (Fig. 2c). In agreement with the above, Twinkle and some of the mtSSB and polG co-fractionate with the cholesterol in the ρ° digitonin pellet flotation fraction, as did ATAD3. Similar to the top-down fractionation, this indicates that a proportion of these nucleoid proteins persists in association with a membrane platform in the absence of mtDNA. The structure and composition of the cholesterol membrane domain at the nucleoid-containing sites also do not depend on respiratory chain complexes or normal cristae formation, which does not occur in ρ° cells^32^. All data so far thus suggest not only that Twinkle is tightly membrane associated even in the absence of mtDNA, as has been previously observed^7^, but that a specialized cholesterol containing membrane platform is responsible for the association of the Twinkle-nucleoid with the inner-membrane.

### ATAD3 knock-down modifies the distribution of cholesterol and the membrane-associated nucleoid

**Figure 3:**
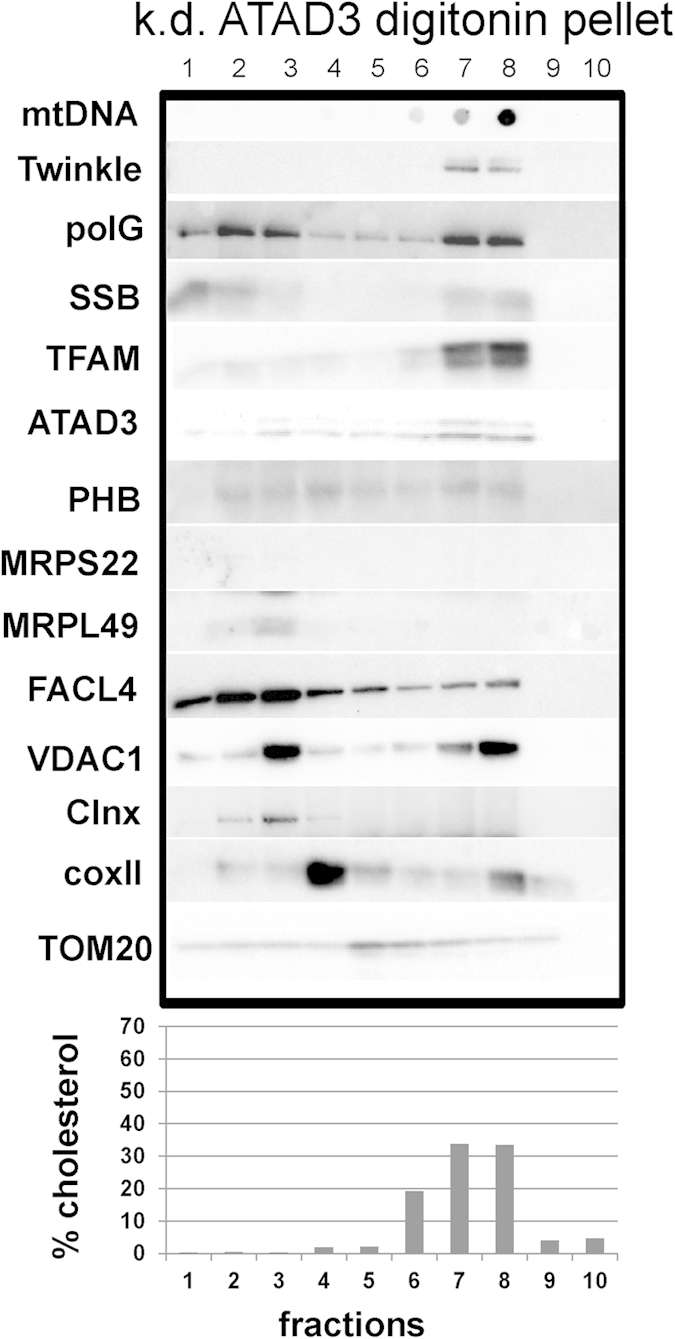
Knock-down of ATAD3 disrupts the cholesterol containing fraction and with it the membrane associated nucleoid. Pellet preparations from digitonin treated purified mitochondria derived from ATAD3 siRNA treated HEK293 cells were separated on a bottom-up flotation iodixanol gradient. The cholesterol and corresponding nucleoid components no longer migrate tightly as one fraction, but are spread evenly over two fractions. mtDNA, mitochondrial DNA; polG, polymerase Gamma; SSB, single-stranded binding protein; TFAM, mitochondrial transcription factor A; PHB, prohibitin, MRPS, mitochondrial ribosomal protein small subunit; MRPL, mitochondrial ribosomal protein large subunit; Clnx, calnexin; coxII, cytochrome oxidase subunit II; TOM20, translocase of outer membrane 20 kDa subunit; VDAC1, voltage-dependent anion channel 1. Protein, DNA and lipid profiles of replicate gradients are presented in SFig. 2.

Although the above data make a case for a specialized membrane platform, with which Twinkle containing nucleoids associate, we cannot at this point discard the possibility that the Twinkle-nucleoid fractionates together with most of the mitochondrial cholesterol by coincidence. Therefore, we sought to modify cholesterol homeostasis in the mitochondrial membrane by decreasing the expression of ATAD3, due to its proposed functions in cholesterol homeostasis and because it co-purifies with proteins involved in lipid metabolism (see *Introduction*). Interestingly, although ATAD3 is associated with nucleoids and is able to bind mtDNA^9^,^10^, it was unaffected in the gradients prepared from ρ° cells compared to controls (Figs 1 and 2). This may be due to a strong connection between ATAD3 and mitochondrial membranes. Indeed, based on biochemical data we have already earlier proposed that ATAD3 is involved in tethering nucleoids and mitochondrial ribosomes to the IM^11^. We prepared bottom-up flotation gradients from HEK293 cells treated with siRNAs against ATAD3. Now, instead of being condensed in a single fraction as observed in controls (Fig. 1), the cholesterol was equally spread out across two fractions in the pellet preparation (Fig. 3 and SFig. 2). Importantly, a concomitant spreading of nucleoid markers was observed. This observation suggests that ATAD3 contributes to the organization of the cholesterol-rich membrane domain. Moreover, it demonstrates that the Twinkle-associated nucleoids are physically coupled to the cholesterol-rich material.

Previous studies have shown that ATAD3 binds D-loop structures *in vitro*, that a fraction of the protein is tightly bound to mtDNA or nucleoids, and that protein-bound mtDNA multimers containing the D-loop region, which are putative segregation intermediates, are dependent on ATAD3^10^. In addition, ATAD3 gene silencing decreases Picogreen staining of mtDNA, which is indicative of an increase in negative super-coiling or relaxed DNA^10^. This is a plausible consequence of the loosening of the nucleoid structure due to disrupted membrane organization, as is evidenced by the gradients after ATAD3 knock-down. Hence, the data presented here strengthen the view that ATAD3 plays a role in nucleoid organisation.

**Figure 4:**
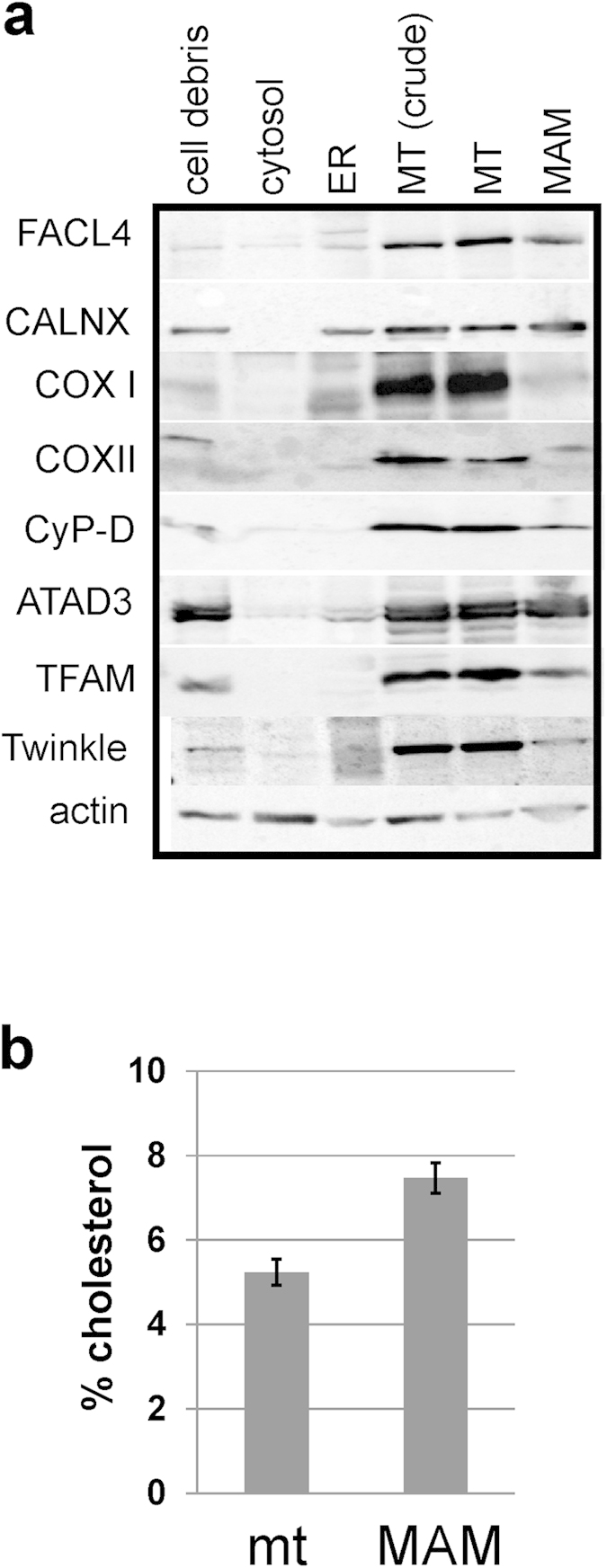
Nucleoid components are detected in purified MAM. Sub-cellular fractionations of HEK293 cells were done according to^29^ Western-blot analyses using different markers to identify cytosolic (actin), ER (CALNX), mitochondrial (coxI, coxII, Twinkle, TFAM, cyclophilin D, ATAD3) and MAM (FACL4) components showed that in HEK293 cells, mitochondria cannot be easily separated from ER membranes. However, some MAM can be freed from mitochondrial membranes as evidenced by the absence of the mitochondrial membrane markers coxI and coxII. Mitochondrial nucleoid components are still detected in this otherwise mitochondria-free MAM-preparation (**a**). The amount of cholesterol (molar percentage of total lipids) in MAM is higher than in purified mitochondria derived from HEK293 cells (mt N =3 ; MAM N = 3) (**b**). ER, endoplasmic reticulum; MT, mitochondria; MAM, mitochondria associated membranes; FACL4, long-chain acyl-CoA synthetase 4; CALNX, calnexin; coxI cytochrome oxidase subunit I; coxII, cytochrome oxidase subunit II; CyP-d, Cyclophilin D; TFAM, mitochondrial transcription factor A.

In addition to maintaining nucleoid and membrane structures, ATAD3 may also be involved in channelling cholesterol to the Twinkle-nucleoid attachment site via ER-mitochondrial junctions^24^. Previously, we found that ATAD3 co-purified with the cholesterol transfer protein STARD9^11^. Recently, it also was suggested that ATAD3 influences cholesterol transfer into mitochondria in steroidogenic cells: ATAD3 was found in MAM of M-10 mouse tumour Leydig cells, and a reduction of ATAD3 levels via RNAi lead to a decrease in hormone dependent progesterone production, which the authors speculated was due to impaired cholesterol import into mitochondria^12^. In our hands, isolation of MAM derived from HEK293 cells also identified ATAD3 and other nucleoid proteins (Fig. 4a), while the cristae membrane markers coxI and II by and large were absent. Vice versa, the MAM marker protein FACL4^29^ was specifically detected in the membrane-nucleoid fraction after flotation, whereas Calnexin, a more generalized ER marker, was not (Fig. 1). Furthermore, also FACL4 followed the redistribution of cholesterol and the Twinkle-nucleoid upon ATAD3 knockdown. This was also the case for the OM protein porin (VDAC1), which is plausible, since it has been detected in MAM previously^33^. Finally, and in agreement with previous studies^34^,^35^ the cholesterol content of purified MAM was higher than that of purified total mitochondria (Fig. 4b). Taken together, these data suggest not only that most of the mitochondrial cholesterol is concentrated at ER-mitochondrial junctions to create a specialized membrane domain, but also that this domain serves as a membrane platform to which Twinkle-nucleoids attach.

### Alterations in mitochondrial ultrastructure via mitofilin depletion do not alter the distribution of cholesterol in mitochondrial membrane flotation gradients

**Figure 5:**
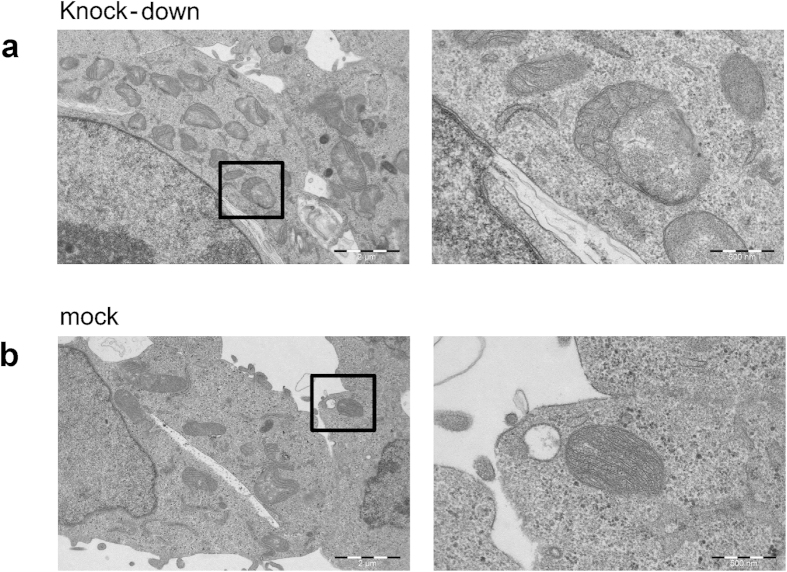
Knock-down of ATAD3 affects mitochondrial membrane structure. TEM images of cells transfected with a scrambled siRNA construct (**b**) and a siRNA against ATAD3 (**a**) reveal changes in the mitochondrial membranes, including abnormal cristae, in the absence of ATAD3 compared with the mitochondria of mock transfected cells.

Knock-down of ATAD3 in Leydig cells led to changes in mitochondrial membrane structure^12^. We likewise examined the morphology of the mitochondria of HEK293 cells after ATAD3 knock-down by electron microscopy. ATAD3 gene silencing lead to various abnormalities in mitochondrial structure, and cristae organization, compared with the mitochondria of mock-transfected cells (Fig. 5 and SFig. 3). Loss of ATAD3 also resulted in membrane-dense onion-like structures (SFig. 3), which have been previously associated with the absence of ATP synthase^36^. This could be a secondary consequence of the loss of respiratory chain components due to the decrease in mitochondrial protein synthesis, which we demonstrated previously^11^. Alternatively, ATP synthase might contribute to mtDNA segregation and organisation, as decreased expression of its components, but not those of respiratory chain subunits, perturbs picogreen-labeling of mitochondrial nucleoids^37^.

**Figure 6:**
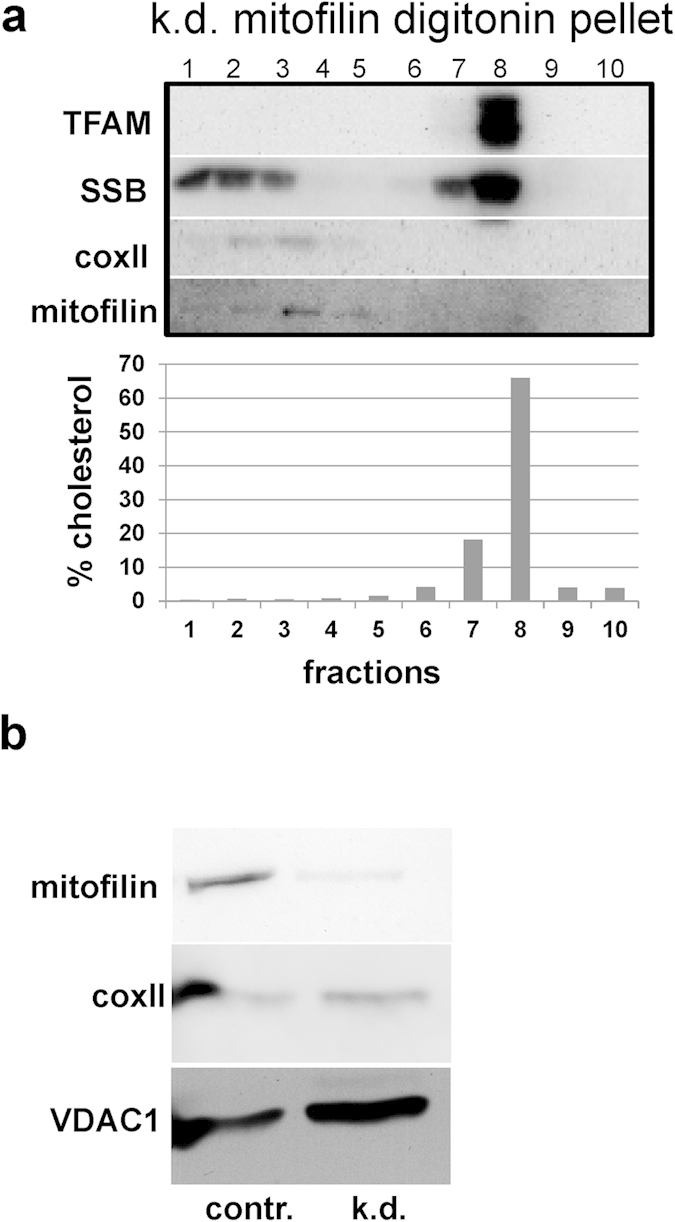
The cholesterol-rich fraction and the associated nucleoid remain intact after the knock-down of mitofilin. Pellet preparations from digitonin treated purified mitochondria derived from mitofilin siRNA treated HEK293 cells were separated on a bottom-up flotation iodixanol gradient (**a**). The majority of the cholesterol and corresponding nucleoid components migrate to the same fraction as in control preparations (Fig. 1). Mitofilin knock-down (**b**). TFAM, mitochondrial transcription factor A; SSB, single-stranded binding protein; coxII, cytochrome oxidase subunit II. Protein, DNA and lipid profiles of a representative gradient.

Mitochondrial cristae formation is in part controlled by mitofilin (or its orthologs like Fcj1 in yeast)^38^,^39^ the knock-down of which also leads to alterations in cristae form and distribution. To investigate if the altered cholesterol distribution after ATAD3 knockdown was an indirect consequence of mitochondrial cristae remodelling, we repeated the flotation analysis following mitofilin knockdown. The results show that even when the amount of mitofilin was reduced significantly the cholesterol domain remained intact in the gradients (Fig. 6). Thus, loss of cristae alone does not seem to affect the integrity of the cholesterol domain. This implies that the formation of the domain is not governed primarily by membrane organization, but in contrast it is maintained by scaffold proteins, such as ATAD3. Our results show that the cholesterol domains exist at sites where the IM connects with the OM and MAM, and that they are likely maintained by protein tethers and lipid traffic between the ER and mitochondria.

## Discussion

Nearly fifty years ago Margit Nass proposed, based on EM images of mouse fibroblast mitochondria, that the mitochondrial DNA (mtDNA) is attached to the inner mitochondrial membrane (IM)^40^. Recently, we reported that the mtDNA helicase Twinkle is tightly membrane associated and, especially in combination with accumulated mtSSB, marks a sub-set of actively replicating mtDNA nucleoids^7^. Here, we propose that the Twinkle-containing mammalian nucleoids attach to a membrane structure that is highly enriched in cholesterol, is held together by ATAD3 and is found in association with ER-mitochondrial junctions. This arrangement would be similar to that suggested for replicating yeast mtDNA nucleoids^31^,^41^,^42^. Our model is based on the following observations: First, in pelleted mitochondrial membranes, which have been treated with Triton X-100 and subsequently subjected to a flotation gradient, the Twinkle-nucleoid fraction migrates to the top of the gradient at low density. This same fraction, when analysed for lipids, is highly enriched in cholesterol and also shows some enrichment of phosphatidylcholine, but not of the non-bilayer forming lipids cardiolipin or phosphatidylethanolamine, which conversely were enriched in higher density fractions. This is plausible, since due to their conical shape cardiolipin and phosphatidylethanolamine are considered unable to pack close to cholesterol molecules in membrane structures. Second, when mitochondria from cells devoid of mtDNA were fractionated with lauryl-maltoside and density-gradient centrifugation as well as in flotation experiments with Triton X-100, Twinkle and several other proteins involved in mtDNA replication persisted to co-fractionate, as they did in the presence of mtDNA. The flotation not only showed that these remnant Twinkle-nucleoids migrated to the same fraction as in cells with mtDNA, but also that this fraction was equally enriched in cholesterol. Of note also is that ATAD3 protein levels in ρ° cells appear unchanged compared to their mtDNA containing counterparts^7^. Third, ATAD3 knockdown modified the distribution of cholesterol and the Twinkle-nucleoid in the flotation gradient equally, demonstrating that the behaviour of cholesterol and the Twinkle-nucleoid are interdependent. Knockdown of mitofilin, which like ATAD3 affects mitochondrial membranes/cristae^34^,^35^ morphology, did not alter the relative distribution of cholesterol or Twinkle-nucleoid markers in flotation gradients compared to controls. Fourth, isolated MAM were found to contain ATAD3 and several tested Twinkle-nucleoid marker proteins, while *vice versa* the Twinkle-nucleoid flotation showed partial co-fractionation with the MAM marker FACL4.

Although we here suggest an arrangement for replicating mammalian nucleoids similar to what has been proposed in *S. cerevisiae*, it is by no means a simple repetition or extrapolation of data published for yeast. In yeast it has been proposed that replicating nucleoids attach to the inner membrane via a membrane-spanning complex that associates with the ER via the so-called ERMES (for *ER-mitochondria encounter structure*)^31^,^40^,^41^. However, although ER-mitochondrial junctions exist in mammals as they do in yeast, the protein composition of these structures, although not fully known, will vary considerably for the simple reason that mammals have no orthologs for the core yeast ERMES proteins^43^, nor do yeast have ATAD3^44^. Some similarities can be drawn though: although yeasts do not have cholesterol, they do have ergosterol, which is structurally similar to cholesterol and is thought to have very similar functions^45^,^46^. The enrichment of mitochondrial ergosterol in yeast nucleoid-membrane preparations has not, to our knowledge, been examined, but it is tempting to speculate that an association similar to what we propose for mammalian nucleoids, involving a specialized membrane environment, also exists in yeast, involving ergosterol. Consistent with this idea, the knockout of ERMES components, which affects mitochondrial nucleoids^31^,^41^ affects mitochondrial membrane lipid homeostasis including mitochondrial ergosterol content^47^.

Here, we pinpoint the location of cholesterol in mitochondrial membranes with a suggested lateral clustering relevant for nucleoid maintenance. Previous work on biochemically isolated mitochondrial membrane fractions has suggested, based on spectroscopic analyses of lipid behaviour, that mitochondrial membrane lipids form domains at IM-OM contact sites, and that the cholesterol to phospholipid ratio is higher on the OM than on the IM side^48^. Our findings further indicate that the majority of the mitochondrial cholesterol is concentrated in specialized structures spanning both the IM and the OM, and not uniformly spread over the inner and outer membranes, and suggest a structural function for mitochondrial cholesterol. This expands our understanding of the role of mitochondrial cholesterol beyond the specialised case of steroid production. Generally, cholesterol is low abundant in mitochondria as well as in the ER network^17^. However, it is considered an important constituent of ER-mitochondrial junctions and MAM^34^,^35^. Although ER-mitochondrial junctions are involved in the formation of stable links between the ER and mitochondria through protein tethers, the linkage does not involve a membrane fusion^24^,^49^. Our results indicate that the vast majority of the cholesterol that we detect in the Twinkle-nucleoid fraction of flotation gradients is mitochondrial: The cholesterol is distributed at the top of the gradient, where Twinkle-nucleoids are also located, suggesting that this cholesterol is integral to the mitochondrial membrane. Also, some of FACL4, which on various occasions has been shown to be a MAM specific marker^29^,^50^,^51^, co-migrates with the nucleoid-membrane fraction. Calnexin in turn, which was shown to distribute also to MAM upon palmitoylation^52^, is absent from this structure. In contrast, the Western blot of isolated MAM (Fig. 4) indicates that calnexin is enriched in them. Since calnexin is a conditional MAM protein but also a more widely distributed ER protein, this suggests that very little ER membrane is in fact associated with the nucleoid-membrane fraction. MAM-associated calnexin has been suggested to be involved in Ca^2+^ signalling^53^, while FACL4 has, among others, a role in lipid biosynthesis. This might explain the specific association of FACL4 with the nucleoid membrane fraction (see below).

The organization of cholesterol is at least in part maintained by ATAD3, which supports the structure of the nucleoid and its membrane environment. ATAD3 and other nucleoid components were detected in purified mitochondria-associated membranes suggesting that nucleoids are juxtaposed with the ER membrane. As commented on in the introduction, various functions for ATAD3 have been proposed, including a function in steroid hormone synthesis via a putative role in cholesterol homeostasis as well as roles in nucleoid organization and mitochondrial translation. Our data reconcile these functions by suggesting that ATAD3 plays a role in mitochondrial cholesterol membrane architecture, perhaps by participating in a barrier that functions in lipid clustering, similar to what has been proposed for prohibitins^22^,^23^. An intimate connection between Twinkle-nucleoids and ER-mitochondrial junctions, which also function as sites of lipid homeostasis, offers a mechanism for membrane growth during mtDNA replication in concert with nucleoid duplication. As such, our results provide a clear demonstration of a specialized membrane organization involving cholesterol, associated with localized (nucleoid) function within the mitochondrial network. We propose that this structure provides a rigid platform and attachment site to enable the coordinated distribution of the nucleoids with the membrane during mitochondrial division.

## Materials and Methods

### Cell culture

HEK293 cells were grown in Dulbecco’s modified Eagle’s medium (DMEM; Lonza BE12-604F) supplemented with 10% FCS (GE Healthcare), in a 37 °C incubator at 8.5% CO_2_. Flp-In T-Rex ρ° cells were grown under the same conditions, but the medium was supplemented with 50 μg/ml uridine (Sigma) and 1 mM Na-pyruvate.

#### RNA-interference

ATAD3 was knocked-down via post-transcriptional gene silencing with 50 nM of Stealth siRNA (custom made by Invitrogen,) with the following pair of complementary oligonucleotides (which targets both ATAD3A and ATAD3B): 5′-UCAAUGAGGAGAAUUUACGGAAGCA-3′; 5′-UGCUUCCGUAAAUUCUCCUCAUUGA-3′ (He *et al*., 2012) using Lipofectamine 2000 (Invitrogen™) in reduced-serum medium, Opti-MEM (Gibco®) according to the manufacturer’s instructions. Cells were first transfected at a confluence of ca 30%, and after 48 hours the RNAi was boosted with a second transfection. 72 hours after the first transfection cells were harvested for mitochondrial isolation or fixed for electron microscopy. Mitofilin was silenced with 15 nM of the following pair of complementary oligonucleotides: 5′-AAUUGCUGGAGCUGGCCUUTT-3′; 5′-AAGGCCAGCUCCAGCAAUUTT-3′. Cells were harvested 48 hrs after transfection. The transfections were controlled with a scrambled siRNA (Negative control, Low GC duplex, Invitrogen).

#### Mitochondrial isolation

Cells were collected and resuspended in hypotonic buffer (4 mM Tris-HCl, pH 7.8, 2.5 mM NaCl, 0.5 mM MgCl_2_ including protease inhibitors (Roche Molecular Biochemicals)) allowed to swell for 6 min and disrupted with 20–25 strokes with a Dounce homogenizer on ice. The suspension was re-isotonised with 400 mM Tris-HCl, 250 mM NaCl and 50 mM MgCl_2_. Nuclei and cell debris were first pelleted by centrifugation at 1200 *g* for 5 min at 4 °C. The low speed centrifugation was repeated once and crude mitochondria were then pelleted from the cytosolic supernatant by centrifugation at 13000 *g* for 10 min at 4 °C. The mitochondria were further purified either by centrifuging on a two-step (1.5 M and 1 M) sucrose gradient for 30 min at 140000 *g* (for gradient fractionation) or on a Percoll gradient (MAM and mitochondrial isolation, see below).

### ER isolation

was performed by centrifuging the cytosolic supernatant first at 20000 *g* for 30 min at +4 °C to pellet plasma membrane contaminants and then at 100000 *g* for 1 h at +4 °C to pellet the ER.

### MAM isolation

was carried out from crude mitochondrial preparations as described in^29^: A suspension of crude mitochondria in re-suspension buffer (225 mM mannitol, 75 mM sucrose and 30 mM Tris-HCl pH 7.4) was layered onto a 8-ml Percoll medium (225 mM mannitol, 25 mM HEPES pH 7.4, 1 mM EDTA and 30% Percoll vol/vol) in 14 ml thin-wall Polyallomer tubes. The tubes were then carefully filled up with re-suspension buffer and centrifuged at 95000 *g* for 30 min at 4 °C. The MAM fraction was then collected from the top of the tube and the purified mitochondria from the bottom of the tube into separate tubes, both were diluted 10× with re-suspension buffer and centrifuged at 6,300 *g* for 10 min at 4 °C. The pure mitochondrial pellet was washed once in re-suspension buffer. The MAM supernatant was transferred to polycarbonate tubes and centrifuged at 100000 *g* for 1 h to pellet pure MAM.

### Flotation gradients of mitochondrial fractions

were carried out as described in^7^: outer and inner membranes of purified mitochondria were partially disrupted with digitonin (Sigma Aldrich) using a digitonin (μg) to protein (μg) ratio of 2/1 in PBS supplemented with protease inhibitors (Roche Molecular Biochemicals). After a 10-minute incubation on ice the mitochondria were centrifuged at 14000 *g* for 10 min at +4 °C to obtain a membrane enriched pellet and associated components and a supernantant containing soluble components. The pellet was resuspended in TN buffer (25 mM Tris-HCl, pH 7.8, 150 mM NaCl, 1 mM DTT, protease inhibitors, 10% sucrose) containing 1% Triton X-100 all at +4 °C and both the pellet and the supernatant (the equivalent to 1 mg of total mitochondrial protein) were mixed with cold Optiprep^TM^ to a final concentration of 42.5%, transferred to MLS-55 centrifuge tubes and overlaid with an 8-step (40, 37.5, 35, 32.5, 30, 27.5, 25, 20, 0%) Optiprep^TM^ gradient prepared in TN containing 1% Triton X-100. The gradients were centrifuged at 100000 *g* for 14 hrs at 4 °C. Fractions (400 μl) were collected from the top and analysed for proteins, DNA and lipids as described below.

### Top-down iodixanol gradient centrifugation of total mitochondrial lauryl-maltoside lysates

was as described in^54^: Purified mitochondria (2 mg/ml) were suspended in 20 mM HEPES pH 7.8, 2 mM EDTA, 210 mM mannitol, 70 mM sucrose and treated with 100 μg/ml of trypsin at room temperature for 30 min. After washing and pelleting the mitochondria three times, they were lysed with 0.4% n-dodecyl-D-maltoside (DDM or lauryl-maltoside) and centrifuged for 10 min at 1000 *g*; the supernatant was loaded on a 20–42.5% iodixanol gradient prepared in 20 mM HEPES pH 7.8, 1 mM EDTA, 50 mM NaCl, 2 mM DTT, 0.05% DDM and protease inhibitors) and centrifuged at 100000 *g* for 14 h. Fractions (500 μl) were collected by punching a small hole into the bottom of the tube.

### Protein analyses

were carried out by western blotting after separation by SDS-PAGE. For the analysis of gradients 20 μl of each fraction was loaded on to the gel. Proteins were transferred from the gel onto a nitrocellulose membrane, which was probed with antibodies against proteins of interest and HRP-conjugated secondary antibodies. ECL reactions were visualized either with X-ray film or a ChemiDoc instrument (Biorad).

### Antibodies

were Twinkle (mouse-monoclonal), kind gift from Prof. Anu Suomalainen-Wartiovaara (see also^7^); TFAM (rabbit-polyclonal), kind gift from Prof. Rudolf Wiesner; ATAD3 rabbit-polyclonal^11^; coxII, Cyclophilin D, POLRMT, PHB1, HSP60 and Mitofilin, from Abcam; polG, coxII, TOM20, VDAC1 and FACL4, from Santa Cruz; MRPL49 and MRPS22, from Proteintech Europe; Calnexin from Cell signaling and SSB from Sigma Aldrich, Atlas.

### mtDNA analyses

were performed by dot blotting. 20 μl of each fraction was suspended in 380 μl of 2× SSC, boiled for 15 min at 95 °C and blotted onto positively charged nylon membranes. The dot blots were hybridized at 48 °C with Dig-labelled probes against cyt*b*, and detected with a dig-antibody using Easy-Hyb (Roche) according to the manufacturer’s instructions. ECL reactions were visualized with a ChemiDoc instrument (Biorad). Alternatively, DNA was extracted from a portion of each gradient fraction, Southern blotted and hybridized to a radiolabelled probe to estimate the level of mtDNA.

#### Lipid analyses

Lipids were extracted from 200 μl of each gradient fraction and from purified total mitochondria and MAM once with 800 μl of milliQ H_2_O and 1 ml of 1:1 chloroform:MeOH and then twice with 1 ml of milliQ H_2_O and 1 ml of 2:1 chloroform:MeOH in glass tubes. After each extraction the tubes were centrifuged at 300 × *g* for 5 min at RT and the organic phase was collected. The solvent was evaporated from the pooled organic phases under an argon stream at 40 °C. The dry lipid film was then re-dissolved in 2:1 chloroform:MeOH and 10% was transferred to a new glass tube for a cholesterol analysis. The remaining 90% was used to determine the amount of total phospholipids in the sample or to detect the cholesterol or total PLs by thin layer chromatography. The solvent was evaporated and the dry lipid films were stored at −20 °C. Cholesterol was quantified using a fluorimetric enzymatic kit, Amplex® Red Cholesterol Assay Kit (Invitrogen) according to the manufacturer’s instructions. The samples were exited with 550 nm and the emission fluorescence was measured at 590 nm with a Varioscan Flash microplate reader (Thermo Scientific). Total phospholipids were determined using a colorimetric inorganic phosphate assay^55^. The absorbance at 797 nm was measured with a Cary 60 spectrophotometer (Agilent technologies). Thin layer chromatography for neutral lipids was performed on HPTLC 60 F_254_ Silica glass plates using hexane:diethyl ether:acetic acid (65:15:1) as the elution solvent and for phospholipids using chloroform:MeOH:H_2_O (25:10:1,1). The lipids were detected with Cu-acetate.

#### Electron microscopy

For electron microscopy samples were prepared from 80–100% confluent cells in a 10 cm culture dish as described in^56^: The cells were washed 2 times with PBS, then fixed in 2% glutaraldehyde and 2% formaldehyde for 30 min at 4 °C and then left in 2% glutaraldehyde in PBS at 4 °C. The embedding, cutting into thin sections and OsO_4_ staining were carried out according to the standard procedure of the Laboratory of Electron Microscopy, University of Turku, Finland. The grids were imaged with a JEOL JEM-1400 plus transmission electron microscope equipped with an 11 Mpx Olympus Quemesa digital camera.

## Additional Information

**How to cite this article**: Gerhold, J. M. *et al*. Human Mitochondrial DNA-Protein Complexes Attach to a Cholesterol-Rich Membrane Structure. *Sci. Rep*. **5**, 15292; doi: 10.1038/srep15292 (2015).

## Supplementary Material

Supplementary Information
