# Corrigendum: Human Mitochondrial DNA-Protein Complexes Attach to a Cholesterol-Rich Membrane Structure

**DOI:** 10.1038/srep17119

**Published:** 2015-12-14

**Authors:** Joachim M. Gerhold, Şirin Cansiz-Arda, Madis Lõhmus, Oskar Engberg, Aurelio Reyes, Helga van Rennes, Alberto Sanz, Ian J. Holt, Helen M. Cooper, Johannes N. Spelbrink

Scientific Reports 5: Article number: 1529210.1038/srep15292; published online: 10192015; updated: 12142015

In this Article, Ian J. Holt is incorrectly listed as being affiliated with ‘Institute for Cell and Molecular Biosciences, Newcastle University Institute for Ageing, University of Newcastle, Newcastle upon Tyne, NE4 5PL, UK.’ The correct affiliation is listed below:

MRC National Institute for Medical Research, London, NW7 1AA, UK

