# Supplementary material for: Human Mitochondrial DNA-Protein Complexes Attach to a Cholesterol-Rich Membrane Structure
Source: Sci Rep. 2015 Oct 19;5:15292. doi: 10.1038/srep15292 (PMC4609938; doi:10.1038/srep15292)
Supplement: Supplementary Information [file srep15292-s1.pdf]

Supplementary Information

Human Mitochondrial DNA-Protein Complexes Attach to a Cholesterol-Rich Membrane Structure

Joachim M. Gerhold[1,3], Şirin Cansiz-Arda[1], Madis Lõhmus[2, 3], Oskar Engberg[2], Aurelio Reyes[4], Helga van Rennes[1], Alberto Sanz[5], Ian J. Holt[5], Helen M. Cooper[2*] and Johannes N. Spelbrink*[1]

[1] Nijmegen Centre for Mitochondrial Disorders, RadboudUMC, Nijmegen, The Netherlands

[2] Faculty of Science and Engineering, Biochemistry. Åbo Akademi, Turku, Finland

[3] Institute of Molecular and Cell Biology, University of Tartu, Estonia

[4] MRC Mitochondrial Biology Unit, Cambridge, UK

[5] Institute for Cell and Molecular Biosciences, Newcastle University Institute for Ageing, University of Newcastle, Newcastle upon Tyne, NE4 5PL, UK

[6] MRC National Institute for Medical Research, London, NW7 1AA, UK

Supplementary figures

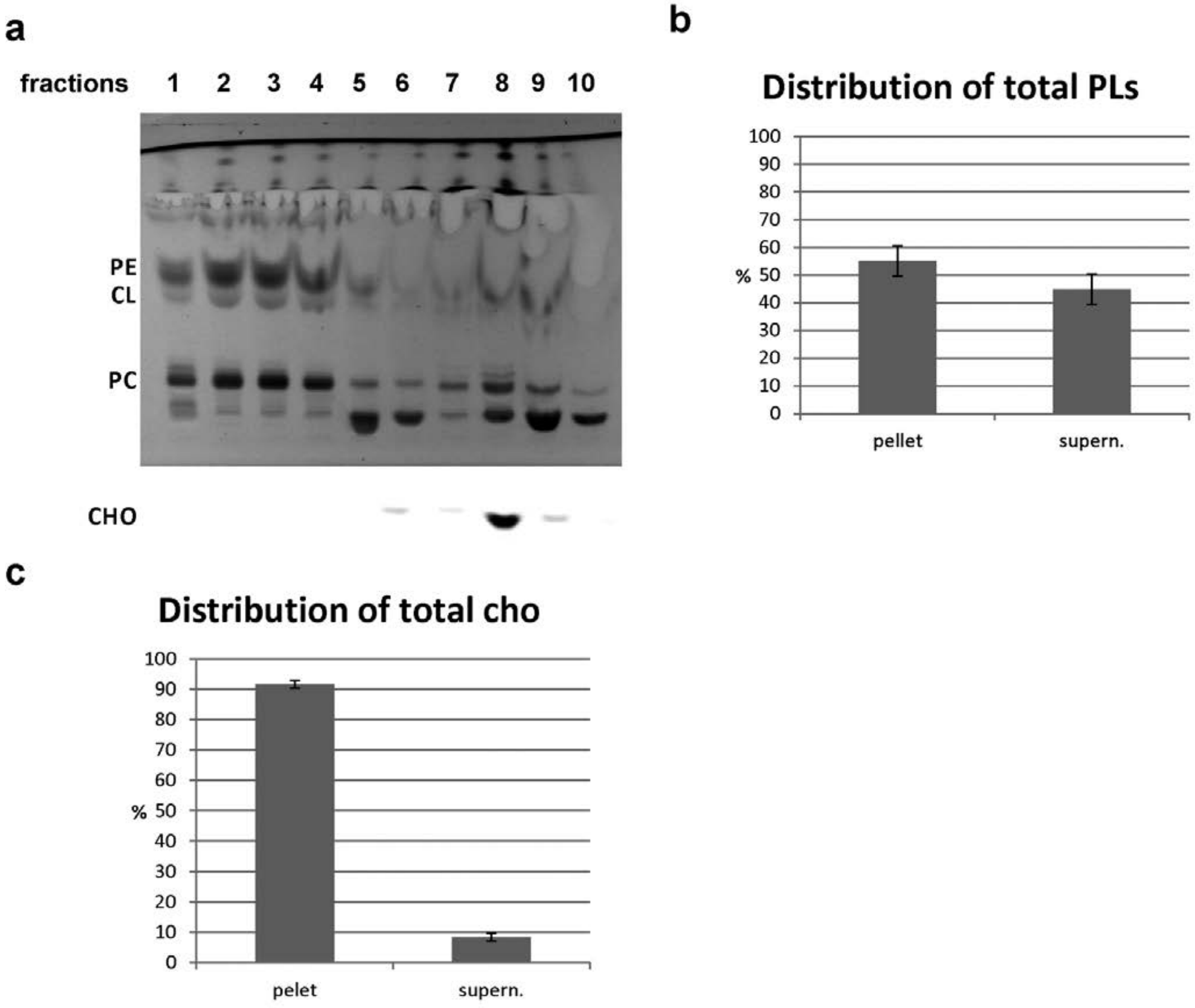

Figure S1 Distribution of different lipid species in a bottom-up floatation gradient of digitonin-derived mitochondrial membrane pellet preparations analyzed by thin layer chromatography (a). Distribution of total phospholipids (b) and cholesterol (c) over the pellet and supernatant gradients (N=3, SEM). PC= phosphatidylcholines, PE = phosphatidylethanolamine, PS = phosphatidylserine, CHO = cholesterol.

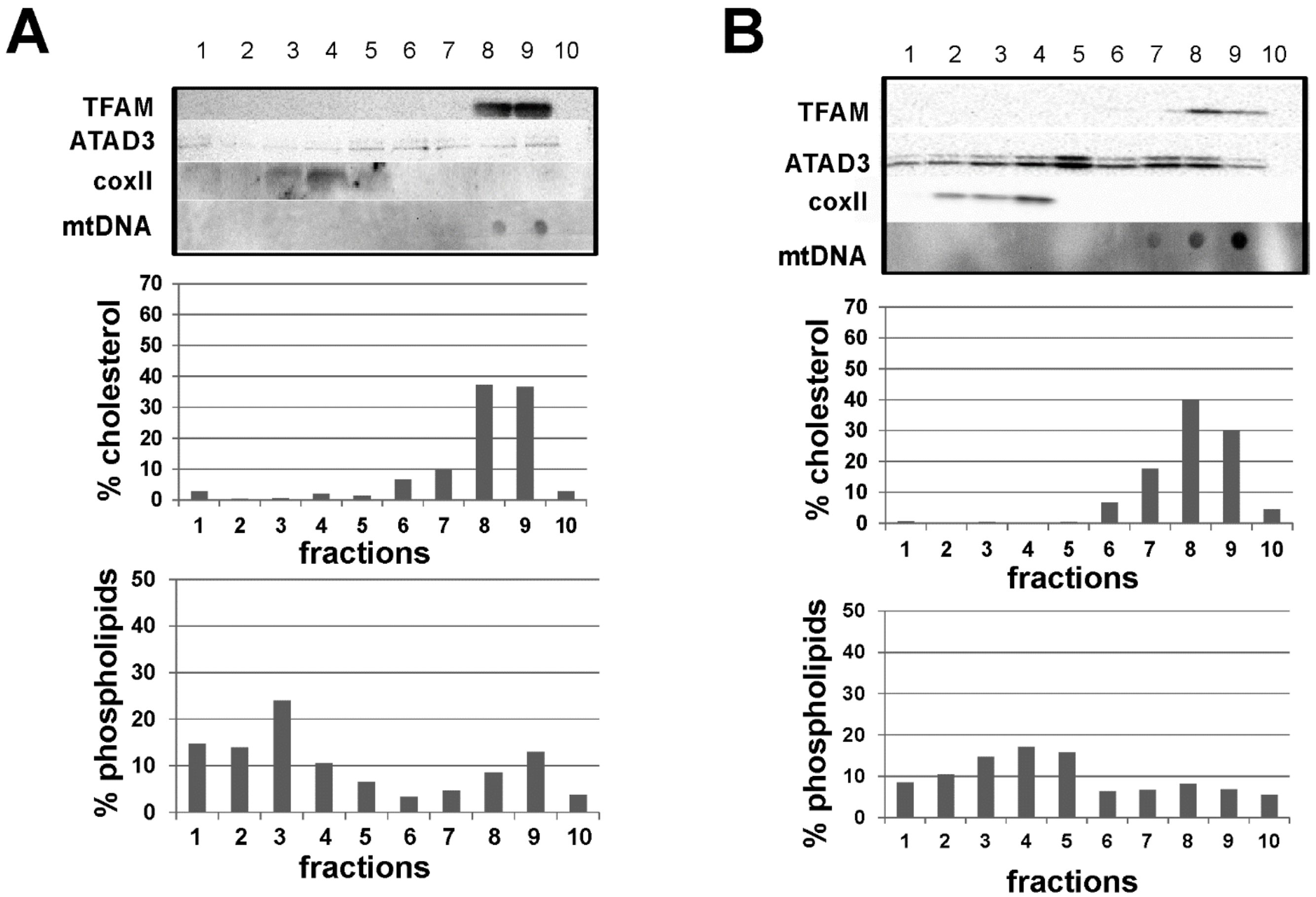

Figure S2 Pellet preparations from digitonin treated purified mitochondria derived from ATAD3 siRNA treated HEK293 cells were separated on a floatation iodixanol gradient. The cholesterol and corresponding nucleoid components no longer sediment tightly in one fraction, but are spread evenly over two fractions, either fractions 8 and 9 or 7 and 8 (Fig. 3). This is likely due to small differences in conditions between preparations, especially to the sensitivity of the membrane to the digitonin treatment, which is likely also influenced by the extent of the ATAD3 knock-down. The phospholipid profile follows the cholesterol profile at the top of the gradient, spreading slightly, otherwise the phospholipids sediments at the bottom of the gradients, similarly to control preparations. mtDNA, mitochondrial DNA; TFAM, mitochondrial transcription factor A; COXII, cytochrome oxidase subunit.

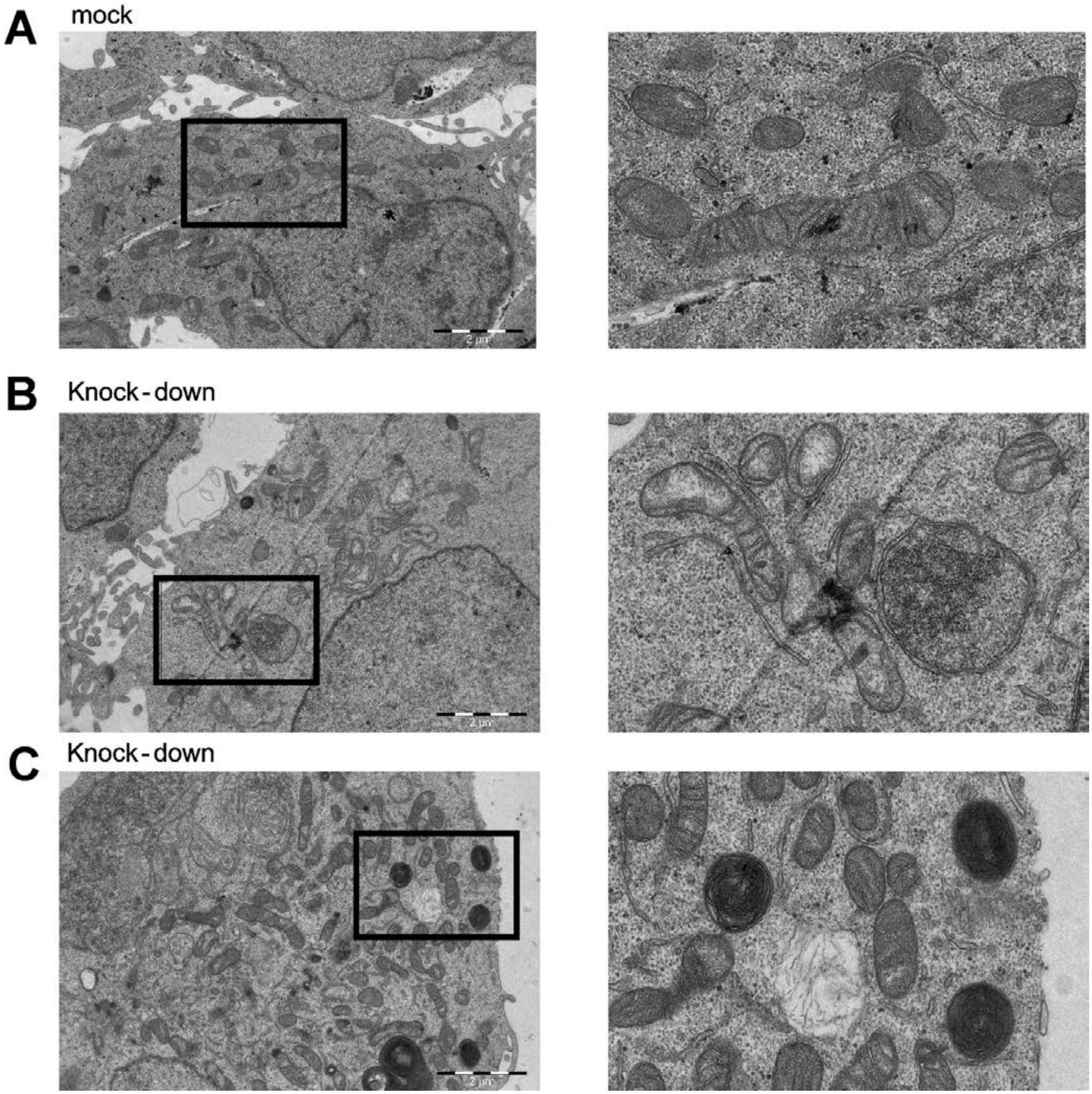

Figure S3 Various abnormalities, including changes in mitochondrial shape and abnormalities in cristae amount, form and distribution as well as membrane-dense onion-like "swirls", are seen in cells after ATAD3 knock-down (B and C) compared with control cells (A).
